# The course of the late-life depression with different immunophenotypes

**DOI:** 10.1192/j.eurpsy.2025.1356

**Published:** 2025-08-26

**Authors:** V. Pochueva, T. Safarova

**Affiliations:** 1 FSBSI Mental Health Research Center, Moscow, Russian Federation

## Abstract

**Introduction:**

According to epidemiological data, about a third of elderly and senile people suffer from mental disorders. The most common are depression and dementia. An urgent problem of modern research is the search for neurobiological correlates of the course and outcomes of late-life depression.

**Objectives:**

To study the outcomes of depression at a late age during a 3-year prospective follow-up in patients with various immunophenotypes.

**Methods:**

A cohort of patients with depressive disorders who were treated in hospital and re-examined in 1 and 3 years. The work carried out a comparative analysis of the course of the disease in patients with different immunophenotypes. The follow-up observation group with immunophenotype A (group 1) included 20 people, 6 men (30.0%) and 14 women (70.0%), median age was 68 years old [62.5; 76.5]. 13 patients (65.0%) were diagnosed with a depressive episode of DE in the framework of recurrent depressive disorder (DDR-F33), 7 patients (35.0%) - in the framework of bipolar affective disorder (BAR-F31). The group with immunophenotype B (group 2) included 31 people, 10 men (32.3%) and 21 women (67.7%), median age was 68 years old [64.0; 72.0]. In 20 patients (64.5%) DDR was diagnosed, in 9 patients (29.0%) - BAR, and in 2 patients (6.5%) – single DE. The patients were examined using clinical, psychometric, immunological and follow-up assessment in 1 year and 3 years.

**Results:**

A comparative study of the course and outcomes of late-age depression in groups of patients with different immunophenotypes, both immediate (1 year) and distant (3 years of follow-up) showed a statistically significant predominance of a favorable course of the disease in patients of group 1 compared with patients of group 2.

In patients of group 1 (in 95% of cases), a favorable course of the disease was noted with the formation of qualitative remissions after 1 year and persisted after 3 years of follow-up. In most patients of the 2nd group, the unfavorable course of the disease prevailed, which was noted both with short-term (1 year - 83.9% of cases) and long-term (3 years - 87.1% of cases) observation. The unfavorable course of the disease in group 2 patients was characterized by a statistically significantly more frequent (p<0.05) formation of incomplete remissions with the preservation of residual depressive disorders, the development of repeated depressive phases on their background according to the type of “double depressions”, as well as cases of chronic depression by 3rd year of observation.

Cases of dementia formation and deaths were noted in this group.

**Conclusions:**

The results of the follow-up observation of patients with late depression indicate that the variants of the further course of the disease are closely related to immunological features. The depressions of the apathetic-adynamic structure, in complete remissions in the anamnesis is a marker of a prognostically less favorable course of the disease.

**Disclosure of Interest:**

None Declared

